# Recall and bias of retrieving gene expression microarray datasets through PubMed identifiers

**Published:** 2010-03-28

**Authors:** Heather A Piwowar, Wendy W Chapman

**Affiliations:** 1 Department of Biomedical Informatics, University of Pittsburgh, Pittsburgh, PA USA

**Keywords:** information retrieval, data sharing, databases, bioinformatics, PubMed, gene expression microarrays

## Abstract

**Background:**

The ability to locate publicly available gene expression microarray datasets effectively and efficiently facilitates the reuse of these potentially valuable resources.  Centralized biomedical databases allow users to query dataset metadata descriptions, but these annotations are often too sparse and diverse to allow complex and accurate queries.  In this study we examined the ability of PubMed article identifiers to locate publicly available gene expression microarray datasets, and investigated whether the retrieved datasets were representative of publicly available datasets found through statements of data sharing in the associated research articles.

**Results:**

In a recent article, Ochsner and colleagues identified 397 studies that had generated gene expression microarray data.  Their search of the full text of each publication for statements of data sharing revealed 203 publicly available datasets, including 179 in the Gene Expression Omnibus (GEO) or ArrayExpress databases.  Our scripted search of GEO and ArrayExpress for PubMed identifiers of the same 397 studies returned 160 datasets, including six not found by the original search for data sharing statements.  As a proportion of datasets found by either method, the search for data sharing statements identified 91.4% of the 209 publicly available datasets, compared to 76.6% found by our search for PubMed identifiers.  Searching GEO or ArrayExpress alone retrieved 63.2% and 46.9% of all available datasets, respectively.  Studies retrieved through PubMed identifiers were representative of all datasets in terms of research theme, technology, size, and impact, though the recall was highest for datasets published by the highest-impact journals.

**Conclusions:**

Searching database entries using PubMed identifiers can identify the majority of publicly available datasets.  We urge authors of all datasets to complete the citation fields for their dataset submissions once publication details are known, thereby ensuring their work has maximum visibility and can contribute to subsequent studies.

## Background

The number of publicly available biomedical research datasets, such as those based on gene expression microarray experiments, continues to increase. The ability to access and process these large datasets enables other scientists to perform their own data driven studies, reduces duplicate data collection, allows the study of issues that require combining multiple datasets, and facilitates the training of future scientists through the analysis of real experimental data. 

To realize these potential benefits, it is necessary that datasets can easily be found when needed. Biomedical databases typically include structured data fields indicating number of data samples, experimental platform and organism and tissue-type or disease of study. The experimental design, controls, and interventions involved are usually described in free-text fields. Unfortunately, the content of these descriptions is often sparse and diverse [1]. As a result, although basic queries of the structured fields can be effective, more complex queries may require pre-processing steps [2] and lack the accuracy required for some applications [3, 4].

Many publicly available datasets are associated with rich annotation outside the database: the published article describing the primary generation and analysis of the data. Centralized biomedical databases often include a “primary citation” field to link to the original published article or articles. This unambiguous link permits a user to query the article metadata, indexing terms, abstracts, or even the full text of the article, and then receive links to datasets relevant to the query. 

The usefulness of Medical Subject Heading (MeSH) indexing terms for annotating gene expression datasets has been described by Butte and colleagues [3, 5-6]. For example, they found that 53% of gene expression microarray datasets in the National Center for Biotechnology Information (NCBI) Gene Expression Omnibus (GEO) database were linked to articles with disease related MeSH terms [3], that control/intervention gene expression data are publicly available for diseases contributing to 30% of all disease-related mortality in the United States [5], and that approximately 10% of microarray experiments in GEO have MeSH terms related to pharmacological substances [6]. We expect that the use of PubMed annotations for dataset retrieval will increase, particularly as combining text and data analysis becomes more common [4, 7-12].

To identify the links between articles and their accompanying datasets, ideally a scientist could simply query PubMed, PubMed Central, or a specialized value added interface (e.g. MedMiner [13], BioText [14], or others [15]) and receive links to related datasets. This is possible within the Entrez network of databases. By appending “AND pubmed_gds [filter]” to any PubMed query, the set of returned articles is limited to those identified as a primary citation in a Gene Expression Omnibus GEO DataSet record. While viewing PubMed results, selecting “GEO Datasets” in the Database dropdown list under “Find related data” in the right-hand column will retrieve the associated datasets. The data can then be explored or downloaded. In many cases, this primary citation query process can be automated. The Entrez databases can be queried through a web service eUtilities interface ( http://www.ncbi.nlm.nih.gov/entrez/query/static/eutils_help.html ). Other databases offer similar web services or application programming interfaces. 

As with any information retrieval strategy, retrieving datasets through their citation field identifiers has limitations. Not all publicly available datasets are submitted to centralized databases, and many are hosted on publisher or laboratory websites. Dataset citation fields are often empty because datasets are frequently submitted to databases before the research article has been published and assigned a PubMed ID. If we use a retrieval strategy based on article metadata, how many datasets are we missing? Are the datasets that are found a representative sample? If not, what are the biases?

To address these questions, in this study we have compared searching for publicly available datasets through statements of data sharing in published articles as reported by Ochsner et al. [16] to searching through queries of centralized databases with article PubMed identifiers. We have focussed on gene expression microarray data, which is expensive to collect, is often shared, has well established data-sharing standards, and is valuable for reuse. The National Center for Biotechnology Information (NCBI) Gene Expression Omnibus [17] (GEO) and the European Bioinformatics Institute (EBI) ArrayExpress [18] databases have emerged as the dominant centralized repositories for sharing gene expression microarray data. Both include fields for primary article citations as PubMed IDs and support querying of those links.

## Methods 

### Reference standard

Ochsner and colleagues [16] manually curated gene expression microarray studies published in 20 journals during 2007. They began with a PubMed filter to identify studies related to gene expression microarray data, reviewed the gene expression articles to identify the subset of studies that generated primary gene expression datasets, and finally searched the full text of the published research articles for statements that the datasets were publicly available either in centralized databases, as supplementary information, or on public websites. 

### Database search for PubMed identifiers

We attempted to replicate the results of Ochsner et al. with a scripted query of gene expression databases. We began with their list of PubMed identifiers for articles identified as generating primary gene expression datasets. We then ran scripts to query the “article submission citation” field of the GEO and ArrayExpress databases with this list of PubMed IDs, and tabulated the datasets thereby retrieved.

We issued scripted queries for GEO and ArrayExpress through their web programmatic interfaces. For example, to query GEO for PubMed IDs 17510434and17603471, we wrote programmatically retrieved the following page:

http://eutils.ncbi.nlm.nih.gov/entrez/eutils/esearch.fcgi?db=pubmed&term=(17510434%5Buid%5D+OR+17603471%5Buid%5D)+AND+pubmed_gds%5Bfilter%5D

and then extracted the <IDList> from the resulting XML. To search ArrayExpress, we issued a query for each PubMed ID:

http://www.ebi.ac.uk/microarray-as/ae/xml/experiments?keywords=**17510434**

http://www.ebi.ac.uk/microarray-as/ae/xml/experiments?keywords=**17603471**

and confirmed the returned pages listed the PubMed ID in the bibliography field. We performed these queries with custom Python scripts.

### Data extraction

For each of the datasets found in centralized databases, we collected the PubMed ID(s), the number of samples in the dataset, the gene expression platform, and the species. We considered the variable for dataset size to be “missing” for datasets shared outside centralized databases because the number of dataset samples was rarely explicitly and consistently stated on journal or laboratory websites.

For each PubMed identifier we collected the name of the journal that published the article, its 2007 Thomson ISI Journal Impact Factor, whether the article was indexed with the MeSH keyword that identifies cultured cells, and whether the article was found by the PubMed “cancer” filter (cancer was the most frequent disease classification for microarray data identified by Butte [3]). We collected PubMed Central citation statistics using the Entrez EUtils web service. 

We determined whether each journal published articles within one specific discipline or had a multidisciplinary scope. We also recorded whether the journal requires authors to include a gene expression microarray database submission accession number in their articles as a condition of publication, following our earlier analysis of journal requirements [19].

If identical datasets were found in more than one location, we made note of this and collected data for the most complete location. Data collection was performed in May 2009 by manual download and with customized scripts (Python 2.5.2 and the EUtils python library [20]).

### Statistical analysis

We calculated the proportion of datasets that were retrievable by the Ochsner search and PubMed identifier queries, using the union of datasets found by either method as a denominator. We estimated the odds that defined subsets of gene expression microarray datasets (those investigating cancer, performed on an Affymetrix platform, involving humans, or involving cultured cells) would be retrieved by querying a database for their PubMed identifiers, relative to the odds they would be found by the Ochsner search but overlooked by the scripted query for PubMed identifiers. Fisher’s exact test was used to determine whether the odds were significantly different than 1.0, with 95% confidence intervals. Histograms and Wilcoxon Rank Sum tests were used to determine whether the distributions of journal impact factors, number of citations, and number of data samples were significantly different between datasets found or overlooked by the PubMed identifier query. Statistics were calculated using the sciplot [21], Hmisc, and Design [22] libraries in R version 2.7.0 [23].

### Availability

Our raw data and statistical R code are available as Supplementary Information and at  http://www.researchremix.org . The raw dataset from Ochsner et al. [16] is available at  http://www.nature.com/nmeth/journal/v5/n12/extref/nmeth1208-991-S1.xls (Archived by WebCite® at  http://www.webcitation.org/5iYQQU4ty ).

## Results

A previous article by Ochsner et al. [16] identified 397 published studies that generated gene expression microarray data. Their examination of data sharing statements revealed that 186 (47%) of these studies had made their datasets publicly available. Fourteen studies had more than one associated dataset (13 studies had two associated datasets, one study had five). The combined 203 datasets were found in a variety of locations: 147 (72%) in the Gene Expression Omnibus (GEO) database, 32 (16%) in the ArrayExpress database, 12 (6%) hosted on journal websites, and 12 (6%) on laboratory websites and smaller online data repositories. Combined, GEO and ArrayExpress housed 179 (88%) of the datasets found by the Ochsner search.

In order to determine the effectiveness of retrieving microarray datasets through an automated search, we attempted to locate these publicly available datasets using scripted queries of centralized microarray databases. We queried the GEO and ArrayExpress databases with the PubMed identifiers of the 397 data producing studies. Our scripted queries returned 160 datasets in total: 132 datasets in GEO and 98 datasets in ArrayExpress, including 70 datasets in both databases (ArrayExpress imports selected GEO submissions).

We compared the retrieval results of the two search strategies: Ochsner‘s search for data sharing statements within the full text of the published studies and our query of centralized databases for PubMed identifiers. As shown in Table 1, the query of databases using PubMed identifiers returned 6 datasets that were overlooked by Ochsner’s search. Data submission dates suggested that one of these six was submitted after publication of the Ochsner paper. Ochsner’s search found 31 datasets in GEO and ArrayExpress that were not found by the PubMed identifier search strategy: 18 of these database entries listed no article citation, 10 listed a different citation by the same research group, two listed incomplete citations lacking a PubMed ID, and one dataset entry included citations to papers by what appears to be a different group of authors.

**Table 1:**
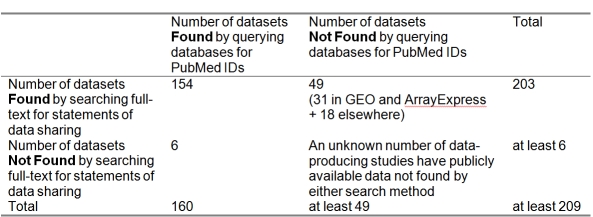
Publicly available datasets found by two retrieval strategies: a search of article full-text for statements of data sharing, and a scripted query of centralized microarray databases for PubMed identifiers.

**Figure 1:**
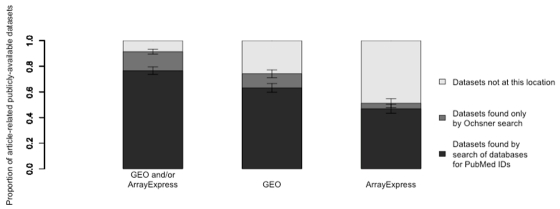
Proportion of article-related, publicly available datasets retrievable by PubMed identifier queries in GEO and ArrayExpress, GEO alone, and ArrayExpress alone (bars indicate 95% confidence intervals of proportions).

The union of retrieval results from both search strategies yielded 209 datasets.  We defined this union as the set “all publicly available datasets” for subsequent analysis. As illustrated in Figure 1, 91% of the 209 publicly available datasets were identified by the Ochsner search, compared to 77% found by queries of GEO and ArrayExpress for PubMed identifiers.  PubMed identifier queries of either GEO or ArrayExpress alone retrieved 63% and 47% of all available datasets, respectively.

Next, we looked at univariate patterns to determine whether the datasets retrieved through our search differed from those found only by the Ochsner search.  The odds that a dataset was about cancer, performed on an Affymetrix platform, involved humans, or involved cultured cells were not significantly different whether the dataset was retrievable through our search method or not (p>0.3).  The recall for datasets from disciplinary journals was similar to the recall from multidisciplinary journals (p>0.1).  In ANOVA analysis, the distribution of species was not significantly different between the two search strategies (p>0.9).  

Datasets found through PubMed identifiers were more likely to be associated with articles in higher impact journals than datasets overlooked by this retrieval method (p=0.01).  Our PubMed identifier search found 92% of datasets from articles published in journals with impact factors greater than 20, 88% of those with impact factors between 10 and 20, and 73% of those with impact factors between 3 and 10.  Journal data sharing policy and journal scope were strongly associated with journal impact factor (p<0.001), but stratifying our dataset by these features only slightly reduced the association between impact factor and recall (minimum p-value for stratified analysis was 0.06). 

**Figure 2:**
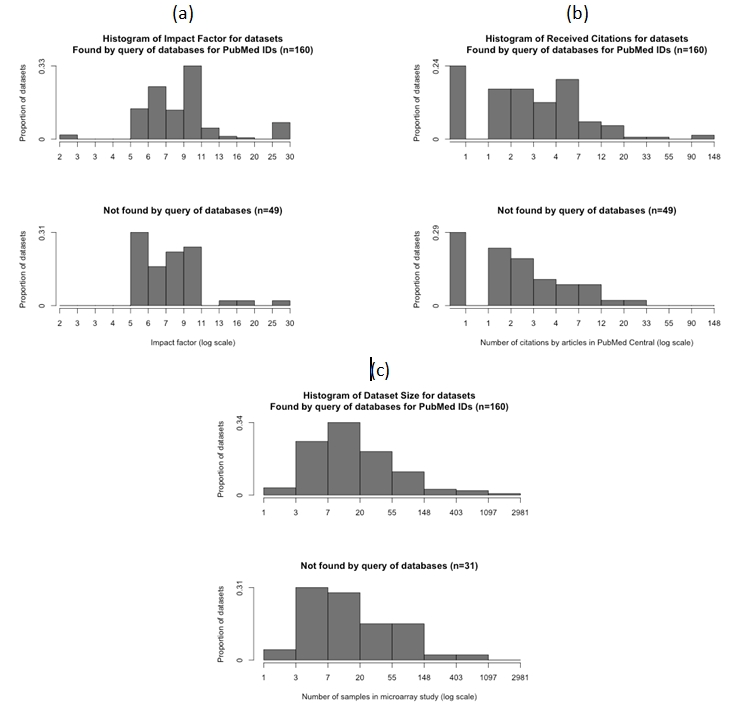
Histograms of datasets found or missed by database queries for PubMed identifiers by (a) impact factor of journal that published the associated study, (b) number of times the associated study was cited by an article in PubMed Central, and (c) number of data points in the dataset.

There was no association between the number of citations received by a study or the study sample size and whether or not the dataset was found by our PubMed identifier query.  Histograms of the impact factors (Figure 2a), citations (Figure 2b), and dataset sample size (Figure 2c) found and overlooked by our query illustrate these patterns.

**Figure 3:**
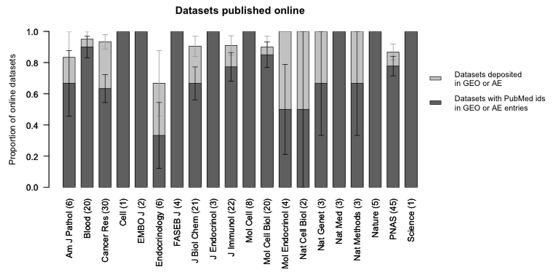
Gene expression microarray datasets published online, by journal.  Light grey bars represent the proportion of online datasets available in the Gene Expression Omnibus or ArrayExpress databases.  Dark grey bars represent the proportion of online datasets that include their publication PubMed identifier in the GEO or ArrayExpress entry, and thus can be found by our retrieval method.  The number of online datasets in our sample follows the journal title, in parentheses.

The ability to retrieve online datasets through PubMed identifiers differed across the twenty journals in our sample, as illustrated in Figure 3, although this difference was not statistically significant in an ANOVA test (p=0.9). 

Finally, we found some evidence that journal policy may be associated with whether a dataset is deposited into a database, complete with PubMed identifier citation.  Our scripted queries found 78% of known publicly available datasets for articles published in journals that require a GEO or ArrayExpress submission accession number as a condition of publication.  This is a higher retrieval rate than we found for publicly available datasets in journals without such a policy (65%), but the difference was not statistically significant (p=0.19).  

## Discussion

In this study we found that scripted queries of centralized microarray databases using PubMed identifiers retrieved 76.6% of all publicly available datasets associated with the publications. The spectrum of datasets was similar to that found by a reference search [16] in terms of array platform, cell source, subject of study, sample size, and study impact.

Dataset retrieval through PubMed identifiers achieved the highest recall when applied to studies from the highest-impact journals. Additional research is needed to understand the reasons behind this finding since it is not fully explained by journal policy or scope, and may have to do with the implementation details of journal policy requirements. The importance of the retrieval bias depends on the intended use of the query results. For example, while there is likely no problem using the query to retrieve datasets for a combination analysis, caution is required when using the results for policy evaluation because query results are not fully representative of all online datasets, 

Our evaluation has several limitations. The evaluation dataset was not chosen randomly and does not contain a representative distribution of journals: in particular, our evaluation subset lacked any journal with an impact factor below 2.5. Also, our reference standard classifications may contain errors, if there exist studies with publicly available data that were identified by neither the Ochsner search nor our PubMed identifier query.

We found that the number of gene expression microarray dataset entries with citation links could be increased by about 25% if all datasets now published on the internet were uploaded to centralized databases, and all primary article citation fields were fully completed. This is consistent with the findings of manual update efforts on the PDB database [24, 25]. We believe encouraging authors and enabling curators to document all link between datasets and research articles is effort well spent. In addition to use in retrieval, a clear relationship between a dataset and its research article allows synergistic documentation, integration for text mining and data mining, and facilitates rewards for publicly sharing data [26, 27].

This study considers the issue of retrieving datasets that are currently available on the internet. As noted by Ochsner et al., data from half of the published gene expression microarray studies does not appear to be publicly shared online [16]. Addressing incentives and policies for increasing the proportion of publicly available datasets is outside the scope of the current study but represents a crucial issue for unleashing the potential of research resources.

## Conclusions

Efficient and accurate dataset retrieval can improve the efficiency of scientific progress, to the extent that it permits detailed review, facilitates integration, and reduces duplicate data collection. Our study suggests that querying gene expression microarray databases for PubMed identifiers is a feasible approach for identifying the majority of publication-related publicly available datasets, particularly when results from GEO and ArrayExpress are combined. The retrieved datasets are representative of all related publicly available datasets. We urge the authors of all datasets to complete the citation fields for their dataset submissions once publication details are known, thereby ensuring their work can have maximum visibility and fully contribute to future scientific studies. 

## Acknowledgements

Funding was obtained from the National Library of Medicine (5T15-LM007059-19 to HP, 5R01-LM009427-01 to WC) and the Department of Biomedical Informatics at the University of Pittsburgh.  The authors thank the editor, Neil Smalheiser, for his valuable feedback.

## Supplementary Material

Additional file 1 rawdata.csv:  Raw characteristics of gene expression microarray studies and their associated datasets. Covariates for 397 gene expression microarray studies, including PubMed identifier, journal of publication, whether associated datasets were found by Ochsner search, whether associated datasets were found by searching databases for PubMed identifiers, database accession numbers, MeSH classifications for species, and array design platform.  A given PubMed ID is listed on multiple rows if it has multiple associated datasets. Click here for file

Additional file 2 statistics.R:  Statistical script.  R code for calculating statistics and graphs in this study paper, given the rawdata.csv file.
Click here for file
